# Supplementary material for: Room-temperature single-photon emission from β-Ga2O3
Source: Nat Commun. 2025 Dec 2;17:247. doi: 10.1038/s41467-025-66953-9 (PMC12783785; doi:10.1038/s41467-025-66953-9)
Supplement: Supplementary file 1 — Supplementary Information [file 41467_2025_66953_MOESM1_ESM.pdf]

Room-temperature single-photon emission from β-Ga_2O_3

Yiming Shi[1,2,#], Zhengchang Xia[2,3,#], Junhua Meng[1,✉], Libin Zeng[2,3], Ji Jiang[2], Zhouxin Li[2,3], Aoxing Wang[2,3], Huabo Yang[2,3], Zhigang Yin[2,3], and Xingwang Zhang[2,3,✉]

[1] School of Physics and Optoelectronic Engineering, Beijing University of Technology, Beijing 100124, P. R. China

[2] State Key Laboratory of Semiconductor Physics and Chip Technologies, Institute of Semiconductors, Chinese Academy of Sciences, Beijing 100083, P. R. China

[3] Center of Materials Science and Optoelectronics Engineering, University of Chinese Academy of Sciences, Beijing 100049, P. R. China

[#] These authors contributed equally: Yiming Shi, Zhengchang Xia

✉ E-mail: jhmeng@bjut.edu.cn (J. M.); xwzhang@semi.ac.cn (X. Z.)

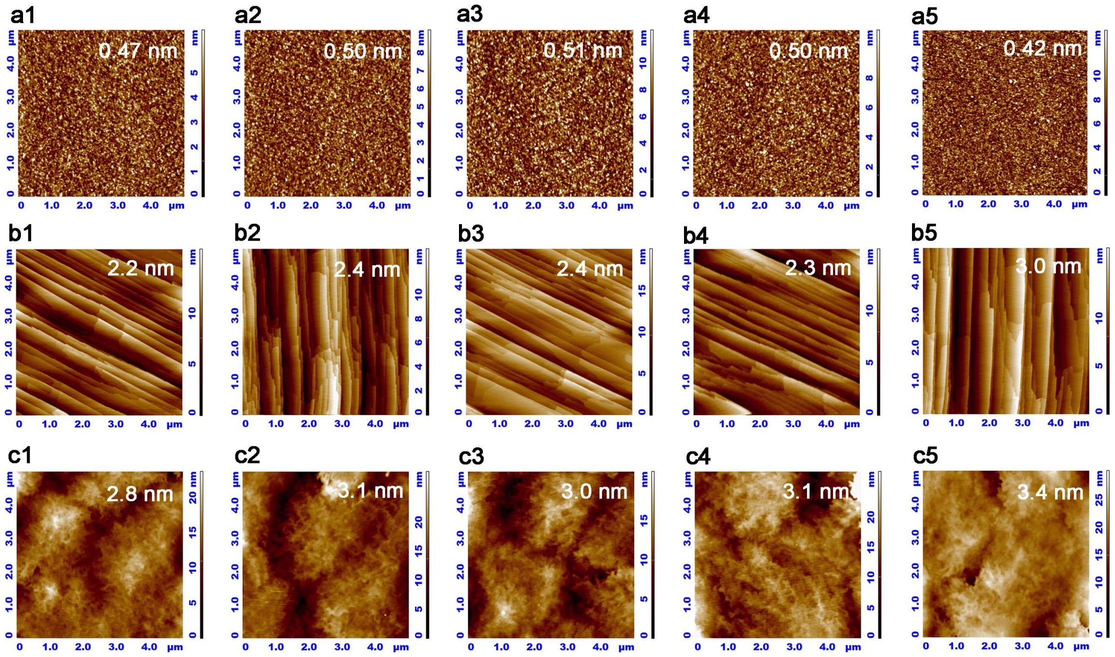

Supplementary Fig. 1 | Atomic force microscopy (AFM) images of the β-Ga_2O_3. a1-a5 Single crystal β-Ga_2O_3 wafer. **b1-b5** Homoepitaxial β-Ga_2O_3 films. **c1-c5** Heteroepitaxial β-Ga_2O_3 films.

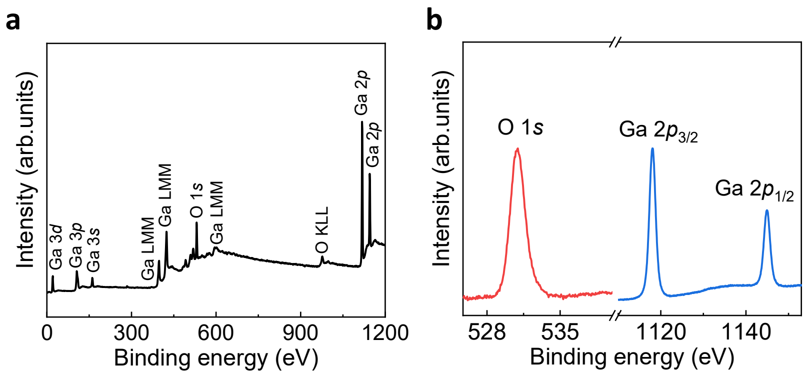

Supplementary Fig. 2 | Chemical composition of the homoepitaxial β-Ga_2O_3 film. a X-ray photoelectron spectroscopy (XPS) full-survey spectrum. **b** XPS spectra of Ga 2*p* and O 1*s* core levels.

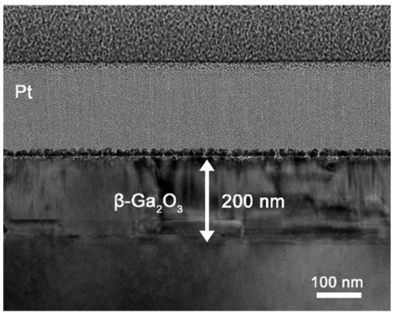

Supplementary Fig. 3 | Low magnification cross-sectional transmission electron microscopy (TEM) image of the homoepitaxial β-Ga_2O_3 film.

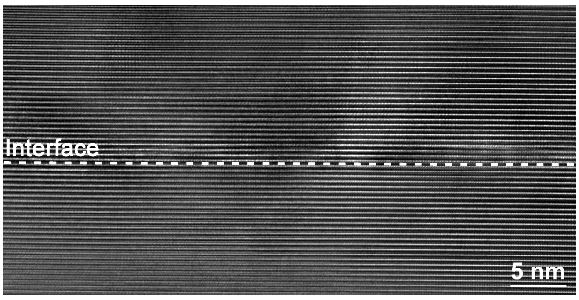

Supplementary Fig. 4 | Global TEM image of the interfacial region for the homoepitaxial β-Ga_2O_3 films. The epitaxy/substrate interface is marked by the dashed line.

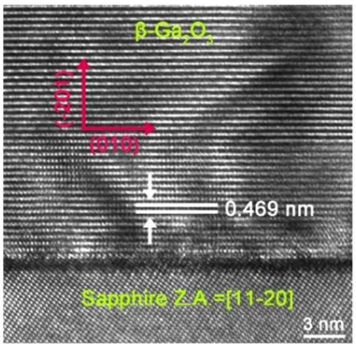

Supplementary Fig. 5 | Cross-sectional high-resolution TEM image of the heteroepitaxial β-Ga_2O_3 film. The epitaxial relationship between the β-Ga_2O_3 epilayer and the sapphire substrate is determined as β-Ga_2O_3 (-201) [010]//sapphire (0001) [1-100].

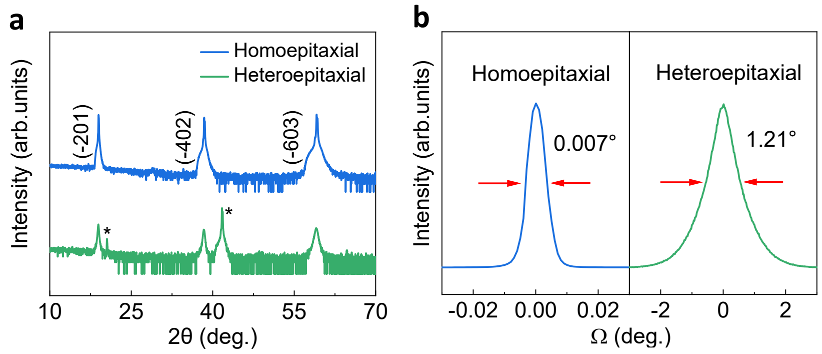

Supplementary Fig. 6 | Crystalline characterization of the homoepitaxial and heteroepitaxial β-Ga_2O_3 films. a X-ray diffraction (XRD) θ-2θ patterns. The asterisks denote the XRD peaks of sapphire substrate. **b** XRD rocking curves of β-Ga_2O_3 (-201) peak.

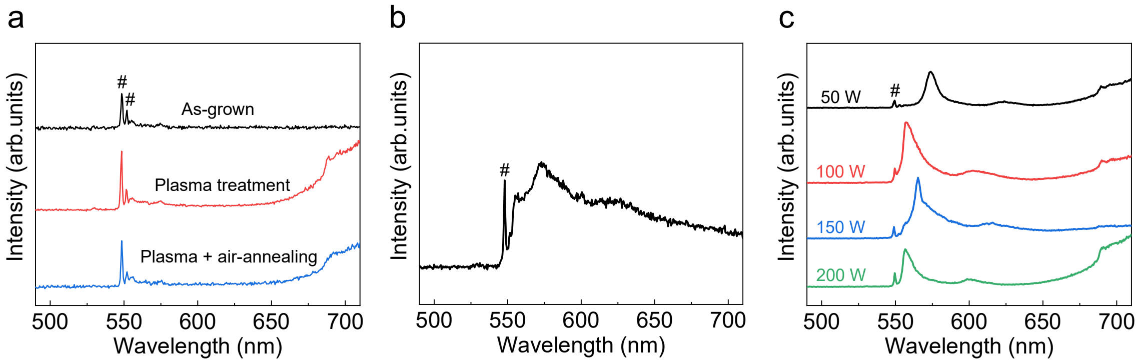

Supplementary Fig. 7 | The photoluminescence (PL) spectra of homoepitaxial β-Ga_2O_3 thin films. a Without treatment and plasma treatment with and without air-annealing. **b** Plasma treatment with vacuum-annealing. **c** Plasma treatment with vacuum-annealing and air-annealing under different plasma powers. The plasma treatment power of 100-150 W is appropriate. The peaks marked by square grids originate from the Raman mode of the β-Ga_2O_3.

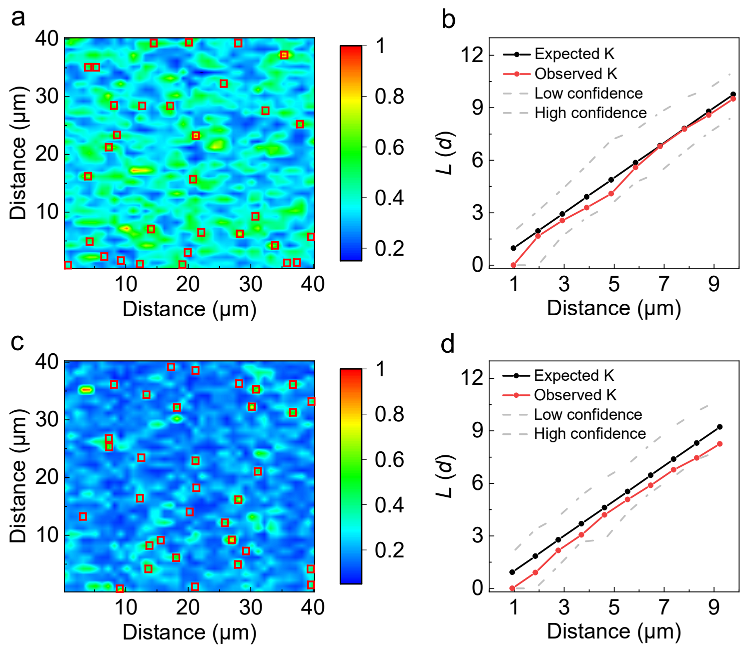

Supplementary Fig. 8 | Distribution of emitters in the homoepitaxial and heteroepitaxial β-Ga_2O_3 thin films. a,c PL intensity maps of the homoepitaxial β-Ga_2O_3 (**a**) and heteroepitaxial β-Ga_2O_3 (**c**) thin films. Red boxes mark isolated emission points. **b,d** The corresponding Ripley's K function of emitters for the homoepitaxial β-Ga_2O_3 (**b**) and heteroepitaxial β-Ga_2O_3 (**d**) thin films. The observed K values consistently fluctuating within the confidence interval, indicating that the emitters are randomly distributed and do not exhibit obvious clustering or dispersion trends.

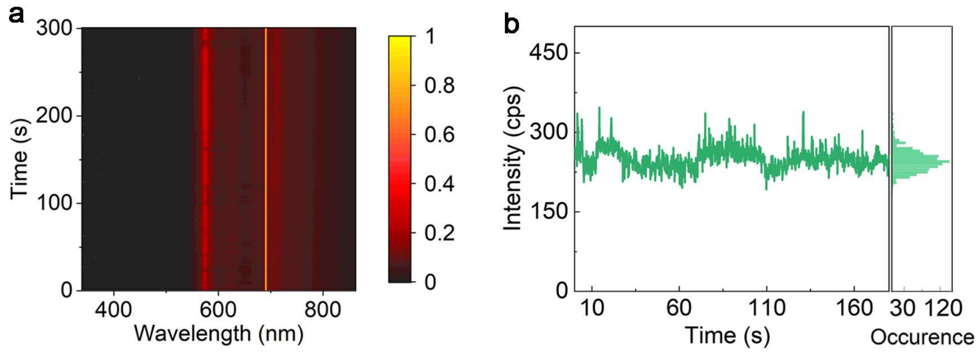

Supplementary Fig. 9 | Photostability of the quantum emitters in the heteroepitaxial β-Ga_2O_3 film. **a** Stability tests of the PL spectra of quantum emitters measured under an excitation power of 5 mW and an integration time of 1 s. **b** Time-dependent PL intensity of the emitter with a sampling time bin size of 200 ms.

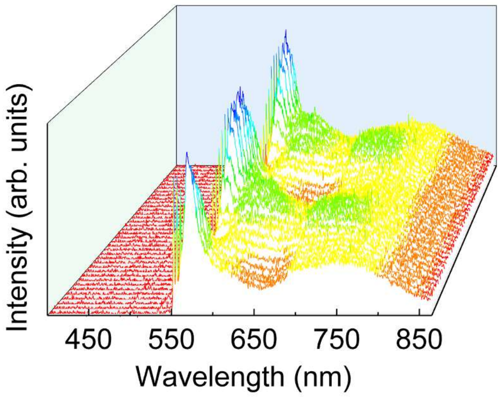

Supplementary Fig. 10 | Waterfall plot of PL spectra at various polarization angles for the emitter in the homoepitaxial β-Ga_2O_3.

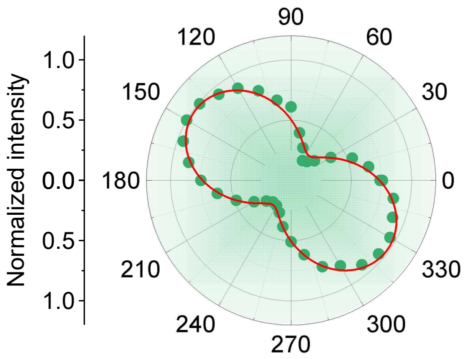

Supplementary Fig. 11 | PL polarization properties of the quantum emitters in the heteroepitaxial β-Ga_2O_3 film. The data are fitted with a $\cos^2(\theta)$ form fit function, yielding the polarization visibility of 68%.

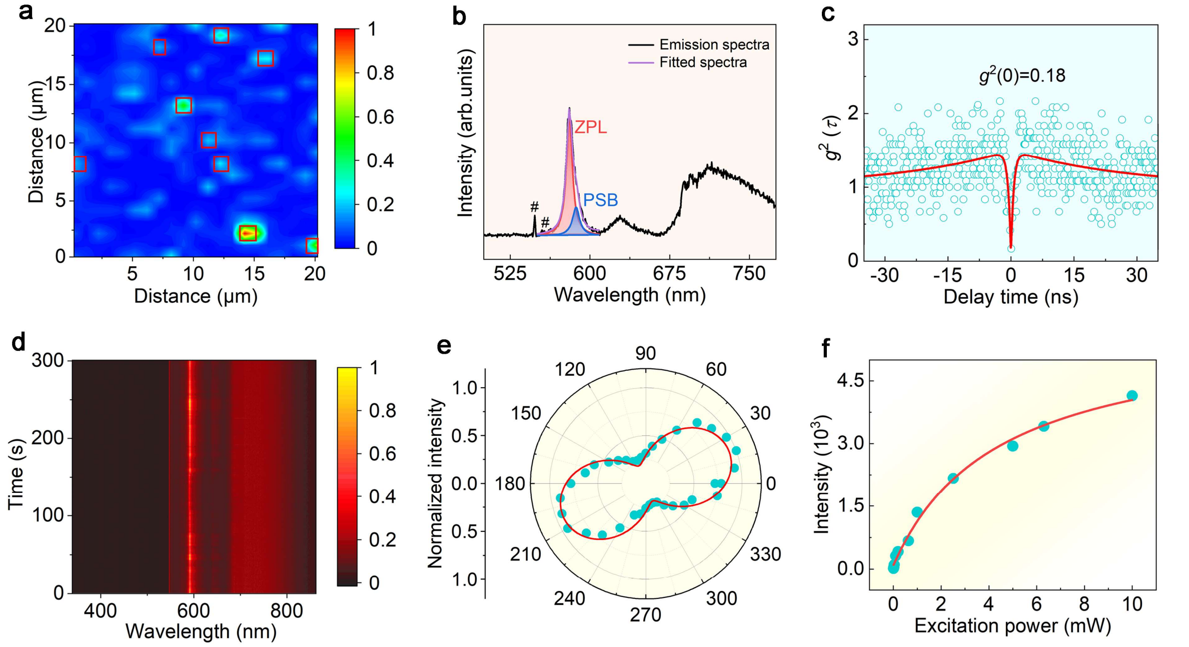

Supplementary Fig. 12 | Single photon emission from single-crystal β-Ga_2O_3 wafer. a Normalized confocal PL intensity maps. Red boxes mark isolated emission points. **b** Representative PL spectrum fitted to obtain the individual weightage of zero-phonon line (ZPL) and phonon sideband (PSB). The peaks marked by square grids originate from the Raman mode of β-Ga_2O_3. **c** Second-order correlation functions $g^2(\tau)$ of the quantum emitter. **d** Stability tests of the PL spectra of quantum emitters measured with an integration time of 1 s. **e** Polar plot of the PL intensity and the polarization angle. The curve is fitted with a $\cos^2(\theta)$ function. **f** Saturation behavior of the PL intensity of quantum emitters along with a theoretical fit.

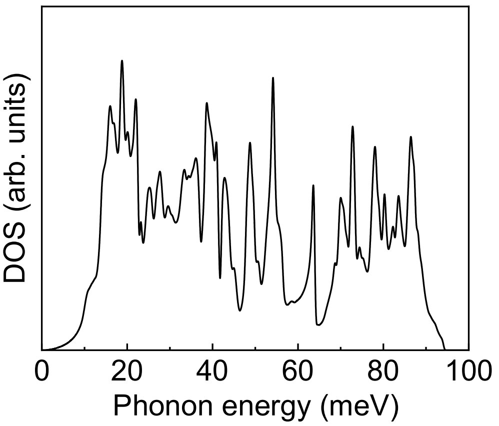

Supplementary Fig. 13 | The phonon density of states (DOS) of bulk β-Ga_2O_3.

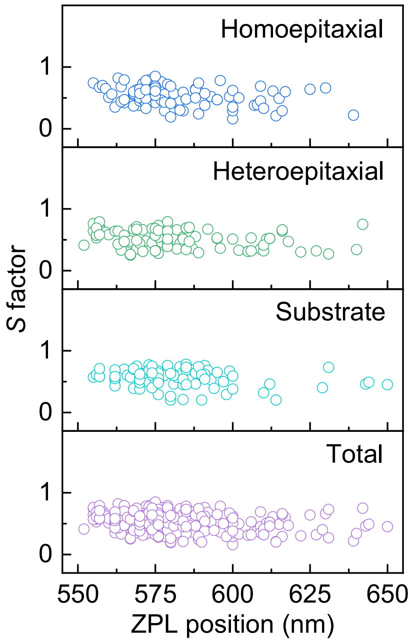

Supplementary Fig. 14 | Statistics of the *S* factor of quantum emitters. The *S* factor represents the number of phonons emitted during vibrational relaxation, and the average value of all *S* factors is ~0.5.

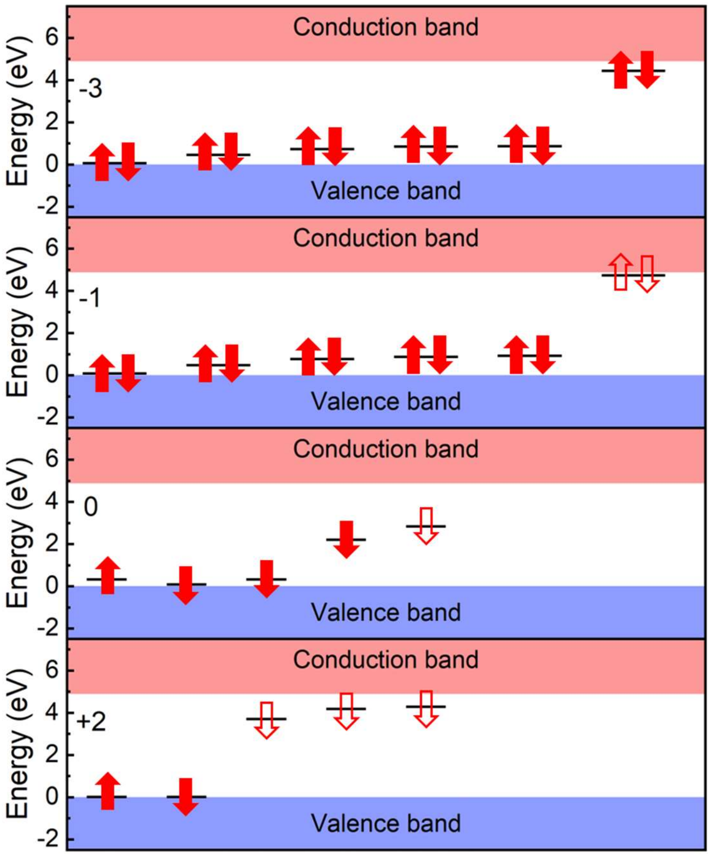

Supplementary Fig. 15 | Kohn–Sham energy levels of V_{GaI}-V_{OIII} defects with different charge states. The occupied and unoccupied states in the bandgap are depicted as red solid and hollow arrows, respectively, where arrow directions signify electron spins.

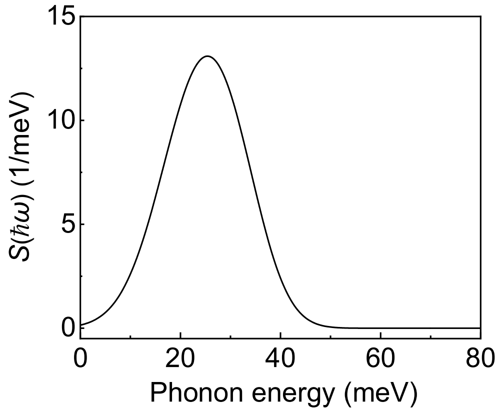

Supplementary Fig. 16 | The spectral function *S*(*ħω*) associated with the spin-doublet optical transition at V_{GaI}-V_{OIII}.

Supplementary Table 1 | Kohn-Sham (KS) levels, ZPL energies, and *S* factors for various vacancy defects with stable charge states. Key properties of various defect including charge state, zero phonon line (ZPL) based on PBE and HSE, position displacement (ΔR), mass weighted displacement (ΔQ), Huang-Rhys factors (S), and magnetic moment (μ). The other parameters were calculated using the PBE functional. Two preliminary screening rules are applied to exclude some defects. The symbol '×' denote the defects without a two-level system, while the symbol '-' denote the defects with calculated S>1.80.

	type	charge	ZPL@PBE (eV)	ZPL@HSE (eV)	ΔR (Å)	ΔQ (Å amu$^{1/2}$)	S	μ
V_O	V_{OI}	0	×	×	×	×	×	×
		+2	×	×	×	×	×	×
	V_{OII}	0	×	×	×	×	×	×
		+2	×	×	×	×	×	×
	V_{OIII}	0	×	×	×	×	×	×
		+2	×	×	×	×	×	×
V_{Ga}	V_{GaI}	-3	×	×	×	×	×	×
		-2	0.49	-	0.120	0.973	1.85	1
		-1	0.63	-	0.132	1.080	2.29	2
		0	0.80	-	0.169	1.385	3.72	3
	V_{GaII}	-3	×	×	×	×	×	×
		-2	0.74	-	0.153	1.252	2.95	1
		-1	0.96	-	0.175	1.436	4.72	2
		0	0.25	2.96	0.097	0.802	1.50	3
V_{Ga}-V_O	V_{GaI}-V_{OI}	-3	×	×	×	×	×	×
		-1	1.85	-	0.169	1.387	3.34	0
		0	0.26	2.71	0.031	0.246	0.10	1
		+2	0.70	-	0.182	1.488	4.58	3
	V_{GaI}-V_{OII}	-3	×	×	×	×	×	×
		-1	1.74	-	0.165	1.348	3.25	0
		0	0.55	-	0.128	1.047	2.13	1
		+2	1.18	-	0.223	1.824	6.32	3
	V_{GaI}-V_{OIII}	-3	×	×	×	×	×	×
		-1	1.85	-	0.159	1.297	3.02	0
		0	0.05	2.34	0.043	0.353	0.28	1
		+2	0.57	-	0.168	1.372	4.07	3
	V_{GaII}-V_{OI}	-3	×	×	×	×	×	×
		-1	2.03	-	0.160	1.315	2.77	0
		0	1.06	-	0.183	1.502	4.91	1
		+2	0.46	-	0.129	1.058	2.43	3
	V_{GaII}-V_{OII}	-3	×	×	×	×	×	×
		-1	2.39	-	0.183	1.497	3.81	0
		0	1.09	-	0.182	1.487	4.34	1
		+2	0.27	2.97	0.097	0.796	1.35	3

		-3	×	×	×	×	×	×
	V_{GaII}-V_{OIII}	-1	2.32	-	0.176	1.438	3.47	0
		0	0.70	-	0.146	1.197	2.32	1
		+2	0.50	-	0.139	1.134	2.47	3
		0	×	×	×	×	×	×
	V_{OI}-Ga_{I}-V_{OI}	+2	1.22	1.63	0.117	0.963	1.25	0
		+4	×	×	×	×	×	×
		0	×	×	×	×	×	×
	V_{OI}-Ga_{I}-V_{OII}	+2	2.02	-	0.125	1.035	2.27	0
		+4	×	×	×	×	×	×
		0	×	×	×	×	×	×
	V_{OI}-Ga_{I}-V_{OIII}	+2	2.18	3.58	0.080	0.663	0.70	0
		+4	×	×	×	×	×	×
		0	×	×	×	×	×	×
	V_{OII}-Ga_{I}-V_{OIII}	+2	1.87	-	0.131	1.082	2.37	0
		+4	×	×	×	×	×	×
		0	×	×	×	×	×	×
	V_{OII}-Ga_{II}-V_{OII}	+2	×	×	×	×	×	×
		+4	×	×	×	×	×	×
		0	×	×	×	×	×	×
	V_{OIII}-Ga_{II}-V_{OIII}	+2	1.29	1.57	0.110	0.908	0.81	0
		+4	×	×	×	×	×	×
V_{O}-V_{O}		0	×	×	×	×	×	×
	V_{OI}-Ga_{II}-V_{OIII}	+2	1.81	-	0.139	1.151	2.78	0
		+4	×	×	×	×	×	×
		0	×	×	×	×	×	×
	V_{OI}-Ga_{II}-V_{OII} d_{OI-OII}=2.92 Å	+2	1.71	-	0.144	1.187	3.03	0
		+4	×	×	×	×	×	×
		0	×	×	×	×	×	×
	V_{OI}-Ga_{II}-V_{OII} d_{OI-OII}=4.01 Å	+2	1.88	-	0.135	1.116	2.92	0
		+4	×	×	×	×	×	×
		0	×	×	×	×	×	×
	V_{OII}-Ga_{II}-V_{OIII} $d_{OII-OIII}$=2.64 Å	+2	1.62	-	0.125	1.031	1.90	0
		+4	×	×	×	×	×	×
		0	×	×	×	×	×	×
	V_{OII}-Ga_{II}-V_{OIII} $d_{OII-OIII}$=2.88 Å	+2	1.51	2.56	0.114	0.943	1.46	0
		+4	×	×	×	×	×	×
		0	×	×	×	×	×	×
	V_{OII}-Ga_{II}-V_{OIII} $d_{OII-OIII}$=4.08 Å	+2	1.92	-	0.143	1.183	3.21	0
		+4	×	×	×	×	×	×
